# Synthesis and Antimicrobial Evaluation of a New Series of Heterocyclic Systems Bearing a Benzosuberone Scaffold

**DOI:** 10.3390/molecules201119701

**Published:** 2015-11-16

**Authors:** Osama I. Abd El-Salam, Ali S. Alsayed, Korany A. Ali, Ahmed A. Abd Elwahab, Abd El-Galil E. Amr, Hassan M. Awad

**Affiliations:** 1 Applied Organic Chemistry Department, National Research Centre, Dokki, Giza 12622, Egypt; osielsalam@gmail.com (O.I.A.E.-S.); kornykhlil@gmail.com (K.A.A.); ahmed_abdelsalam@hotmail.com (A.A.A.E.); 2 Chemistry Department, Al-Azhar University, Nasr City, Cairo 1435, Egypt; ali.sief1980@hotmail.com; 3 Department of Pharmaceutical Chemistry, Drug Exploration & Development Chair, College of Pharmacy, King Saud University, Riyadh 11451, Saudi Arabia; 4 Chemistry of Natural and Microbial Products Department, Pharmaceutical Industries Division, National Research Centre, 33 El Buhouth St. (Former El Tahrir St.), Dokki, Giza 12622, Egypt; awadmhassan@yahoo.com; 5 Deanship of Preparatory Year, Al jouf University, AL Jouf, Skaka 2014, Saudi Arabia

**Keywords:** benzosuberone, nitro-benzosuberone, thioamide, thiophene, thiadiazole, antimicrobial activity

## Abstract

A series of novel benzosuberone derivatives were synthesized and evaluated as antimicrobial agents by using substituted benzosuberone derivatives **1a**,**b** as starting materials. Treatment of **1a**,**b** with phenyl isothiocyanate in dimethylformamide was followed by treatment with cold HCl solution to afford the thioamides **4a**,**b**, which was reacted with methyl iodide to obtain methylated products **5a**,**b**. Cyclocondensation of **4a**,**b** with chloroacetone **6** and phenacyl chloride **7** gave the corresponding thiophene derivatives **9a**–**c**. Reaction of **4a**,**b** with *C*-acetyl-*N*-arylhydrazonoyl chlorides **14a** and **14b** in boiling EtOH in the presence of triethylamine, afforded the corresponding 1,3,4-thiadiazoline derivatives **16a**–**d**. The thioamides **4a**,**b** were reacted with *C*-ethoxycarbonyl-*N*-arylhydrazonoyl chlorides **18a**,**b** which afforded 1,3,4-thiadiazoline derivatives **19a**–**d**. The benzosuberones **1a**,**b** were treated with 3-mercaptopropanoic acid to give compounds **21a**,**b**, which were cyclized to tricyclic thiopyran-4(5*H*)-one derivatives **22a**,**b**. The latter compounds **22a**,**b** were reacted with 3-mercaptopropanoic acid to give compounds **23a**,**b**, which were cyclized tetracyclic ring systems **24a**,**b**. Finally, compounds **24a**,**b** were oxidized using hydrogen peroxide under reflux conditions to afford the oxidized form of the novel tetracyclic heterogeneous ring systems **25a**,**b**. The newly synthesized compounds were screened for antimicrobial activities. The structures of new compounds were characterized by ^1^H-NMR, ^13^C-NMR, IR, and EI-MS.

## 1. Introduction

Thiazolopyrimidine derivatives were studied as potential drug candidates with biological activities [1]. In a previous work, we reported that certain of our newly substituted heterocyclic compounds exhibited androgen receptor antagonists and anticancer activities [2,3]. Also, substituted and condensed cycloalkanones derivatives are of special interest for the preparation of potentially bioactive compounds as they possess anti-inflammatory, anti-convulsant, anti-pyretic, anti-tumor, and anti-ulcer activities [4]. Benzosuberone moiety is the main scaffold in tricyclic antidepressant drugs such as noxiptiline and amitriptyline (the analogues of imipramine), which mostly affect the central nervous systems [5,6]. On the other hand, heterocyclic sulfur compounds are of special interest in modern medicinal chemistry. For example, thiophene and thiadiazole derivatives are a well-known class of biologically active basic compounds for a large number of new drugs [7,8,9]. In view of these observations and in continuation of our current interest in the synthesis of poly-substituted heterocycles for biological evaluations [10,11,12,13,14], the present work was planned to prepare some new benzosuberone derivatives bearing thiadiazole and thiophene moiety, in addition to synthesis of new tricyclic and tetracyclic compounds bearing the benzosuberone scaffold. The newly synthesized compounds were investigated as antimicrobial agents.

## 2. Results and Discussion

### 2.1. Chemistry

**Scheme 1:**
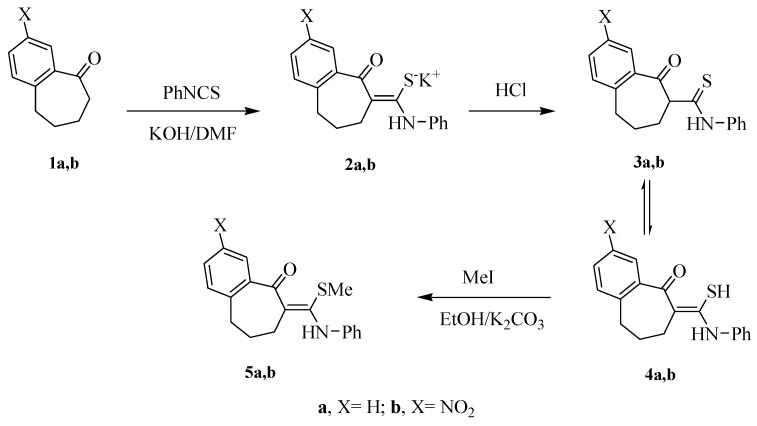
Synthetic routes for compounds **4a**,**b** and **5a**,**b**.

Thioamide and thioanalide intermediates are considered to be a category of versatile intermediates that provide building blocks in the synthesis of poly-substituted thiophene and thiadiazole derivatives [15,16]. Preparation of benzosuberone derivatives substituted with thioamide and thioanalide was achieved by the treatment of the benzosuberone derivatives **1a**,**b** with phenyl isothiocyanate in dimethylformamide, in the presence of potassium hydroxide, followed by treatment with cold HCl solution to afford the thioamide derivatives **4a**,**b** with 81% and 79% yields, respectively (Scheme 1). The ^1^H-NMR spectrum of the thioamide **4a** as an example, revealed the presence of multiple signals at 1.90 ppm and two triplet signals 2.41–3.20 ppm characteristic for 3CH_2_ protons, in addition to a broad signal at 11.50 ppm due to NH proton. The mass spectrum of compound **4a** revealed a peak at *m/z* 295 corresponding to its molecular ion peak. Treatment of the thioamide derivatives **4a**,**b** with methyl iodide in EtONa solution afforded the methylated products **5a**,**b** (Scheme 1). The ^1^H-NMR spectrum of compound **5a** displayed a new singlet signal at 3.08 ppm characteristic for *S*-methyl group.

**Scheme 2:**
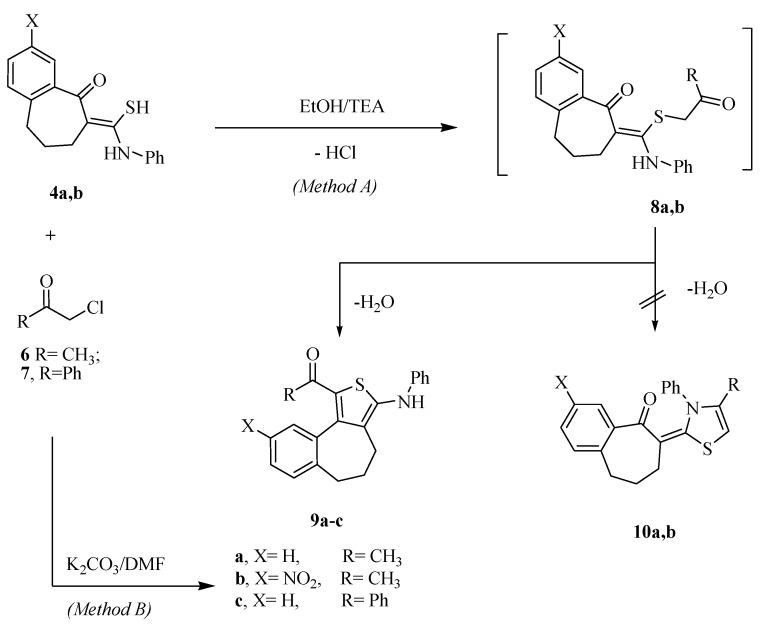
Synthetic routes for compounds **9a**–**c**.

Cyclocondensation of the thioamide derivatives **4a** and **4b** with chloroacetone **6** and phenacyl chloride **7** in refluxing EtOH, in the presence of a catalytic amount of triethylamine furnished, in each case, was only one isolated product. The identities of the isolated products were assigned as the thiophene derivatives **9a**–**c** rather than the isomeric 1,3-thiazoles **10** (*Method A*, Scheme 2), on the basis of their spectral data. The IR spectrum of compound **9b**, as an example of the synthesized compounds, showed the presence of NH and carbonyl bands at 3428 and 1654 cm^−1^, respectively. Moreover, the ^1^H-NMR spectrum of compound **9b**, revealed multiplets at 1.72 and 2.35–2.59 ppm corresponding to 3CH_2_ groups, in addition to signals at 3.30 and 9.53 ppm corresponding to CH_3_ and NH protons, respectively. Compound **9c** was also prepared using thioamide derivative **4a** and phenacyl chloride **7b** in DMF in the presence of potassium carbonate at room temperature (Method B, Scheme 2). The product **9c** from the later method was identical in all respects with the previously obtained authentic sample **9c**.

**Scheme 3:**
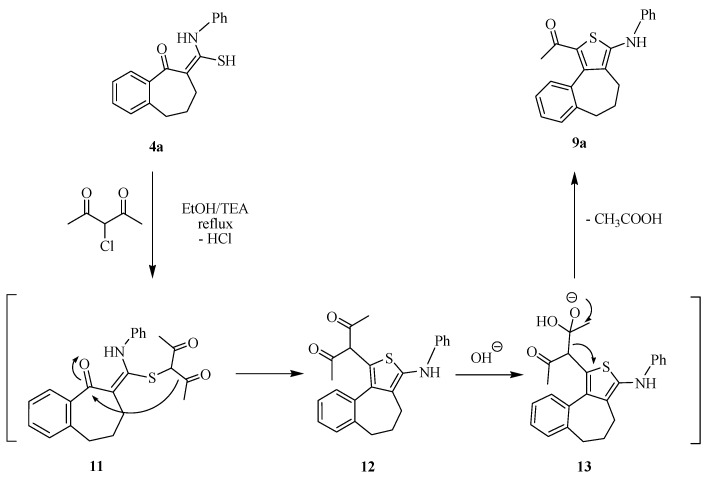
Mechanism of formation compound **9a**.

Further evidence for the proposed structure of compound **9a** was obtained by an independent synthesis via treatment of thioamide derivative **4a** with 3-chloroacetylacetone in refluxing EtOH, in the presence of a catalytic amount of triethylamine. The obtained product **9a** identical in all respects (mp, TLC, and spectra) with that obtained from the reaction of the thioamide derivative **4a** with chloroacetone. A reasonable mechanism of the latter reaction is outlined in Scheme 3, the addition of the haloketone to **4a** with the elimination of HCl gave **11** followed by intramolecular cyclization to give **12**, which under the effect of hydronium ion gave **13** and elimination of acetic acid gave **9a**.

**Scheme 4:**
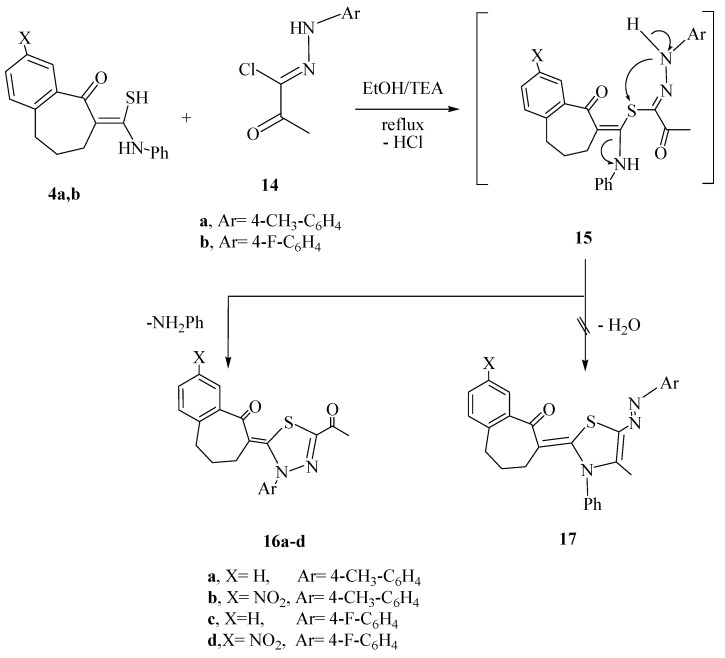
Synthetic routes for compounds **16a**–**d**.

We have investigated the behavior of the thioamide derivatives **4a**,**b** toward hydrazonoyl halide derivatives to prompt our synthetic strategy toward new heterocyclic systems attached to the benzo-suberone scaffold. Thus, treatment of the thioamide derivatives **4a** and **4b** with *C*-acetyl-*N*-arylhydrazonoyl chlorides **14a** and **14b** in boiling EtOH in the presences of triethylamine, afforded in each case only one isolated product. The identities of the isolated products were assigned as the 1,3,4-thiadiazoline derivatives **16a**–**d** rather than the arylhydrazono-thiazole derivative **17** on the basis of their spectroscopic data (Scheme 4). For example, compound **16c** showed characteristic IR bands at 1671, 1653 cm^−^^1^ due to two carbonyl groups. The ^1^H-NMR spectrum of **16c** revealed multiplets at 1.53, 2.38–2.46 ppm due to the protons of 3CH_2_ groups and signal at 3.43 ppm due to the acetyl protons. In addition, its mass spectrum revealed a peak at *m/z* 380 corresponding to its molecular ion peak.

**Scheme 5:**
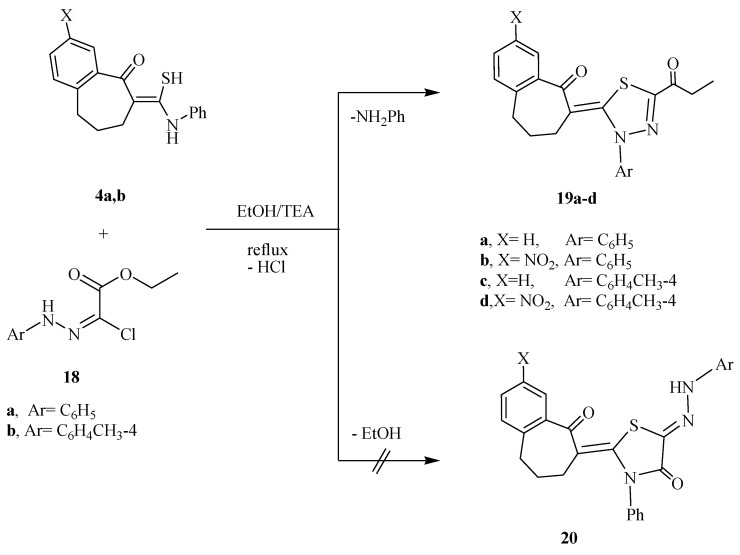
Synthetic route for compounds **19a**–**d**.

In a similar manner, the thioamides **4a** and **4b** were reacted with *C*-ethoxycarbonyl-*N*-arylhydrazonoyl chlorides **18a** and **18b** under the same reaction condition and afforded 1,3,4-thiadiazoline derivatives **19a**–**d** rather than the thiazole-4-one derivative **20** on the basis of their spectroscopic data (Scheme 5). For example, compound **19a** showed characteristic IR bands at 1718, 1675 cm^−^^1^ due to 2C=O groups. The ^1^H-NMR spectrum of **19a** was revealed triplet and quartet signals at 1.71 and 4.20 because of methyl and methylene of the ester group, in addition to multiplets in the region 2.36–2.8 ppm due to the protons of 3CH_2_ groups. The mass spectrum of compound **19a** revealed a peak at *m/z* 392 corresponding to its molecular ion.

**Scheme 6:**
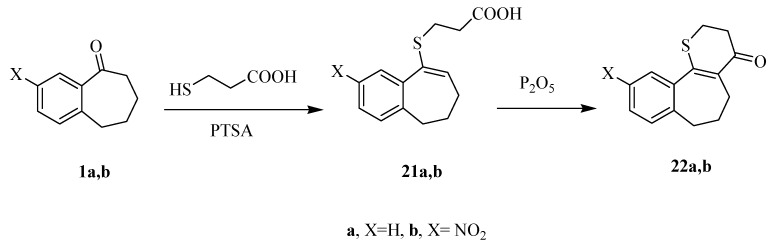
Synthetic routes for compounds **21a**,**b** and **22a**,**b**.

The reaction of **1a**,**b** with 3-mercaptopropanoic acid in refluxing benzene, in the presence of 4-toluenesulfonic acid (PTSA), results in compounds **21a**,**b** which undergo interamolecular cyclization under reflux temperature using phosphorus pentoxide to afford the novel tricyclic thiopyran-4(5*H*)-one derivatives **22a**,**b** (Scheme 6). The structures of the latter products were established on the basis of their elemental analysis and spectral data.

**Scheme 7:**
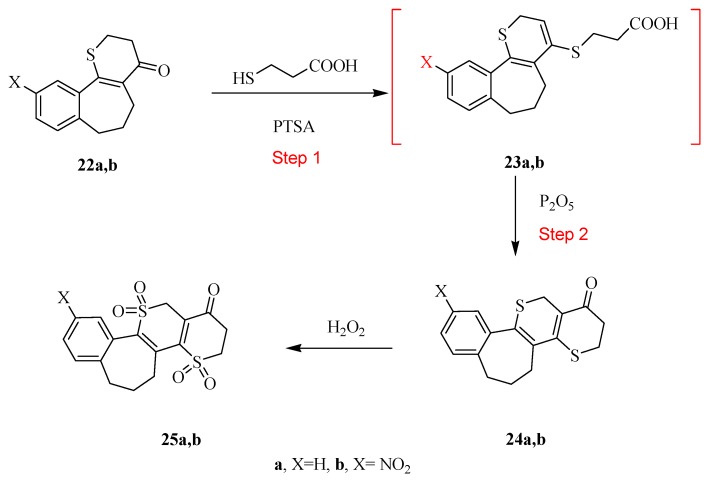
Synthetic routes for compounds **24** and **25**.

Treatment of the thiopyran-4(5*H*)-one derivatives **22a**,**b** in step 1 with 3-mercaptopropanoic acid in refluxing benzene, in the presence of 4-toluenesulfonic acid (PTSA), afford the non-isolated intermediates **23a**,**b** that undergo intramolecular cyclization under reflux temperature using phosphorus pentoxide to afford the novel tetracyclic ring systems **24a**,**b** (Scheme 7). The structures of compounds **24a**,**b** were established on the basis of their elemental analysis and spectral data. The IR spectrum of compound **24a** showed the presence of the carbonyl band at 1692 cm^−1^. The ^1^H-NMR spectrum of **24** revealed multplites at 2.25–2.50 and 3.25–3.38 ppm corresponding to 5CH_2_ groups, in addition to the signal at 3.73 ppm corresponding to CH_2_ of the thiopyran protons.

The later products were oxidized using hydrogen peroxide under reflux condition to afford the oxidized form of the novel tetracyclic derivatives **25a**,**b** (Scheme 7).

Due to the oxidation of compounds **24a**,**b** to compounds **25a**,**b,** the carbonyl band in the IR spectrum of compound **25a** for example shifted to 1695 cm^−1^. The ^1^H-NMR spectrum of compound **25a** revealed multplites near 1.76, 2.15, 2.63 and 3.27 ppm corresponding to 5 CH_2_ groups, in addition to signal at 2.63 ppm corresponding to CH_2_ of the dioxothiopyran protons.

### 2.2. Antimicrobial Screening

The *in vitro* antimicrobial activity of the synthesized compounds was screened against three gram-positive bacteria (*Bacillus subtilits*, *Staphylococcus aureus* and *Enterococcus faecalis*) and three gram-negative bacteria (*Escherichia coli*, *Pseudomonas aeruginosa* and *Proteus sp.*), yeast (*Candida albicans*) and filamentous fungi (*Aspergillus niger* and *A. flavus*). The tested products have shown a strong to moderate effect against most of the tested pathogens.

Most of the compounds showed a moderate inhibitory effect against Gram-positive bacteria (*Bacillus subtilits*, *Staphylococcus aureus* and *Enterococcus faecalis*) except compounds **25b** and **22a**, which showed strong inhibition effect in comparison with the standard antibiotics used (Table 1). On the other hand, five compounds **25b**, **9c**, **19b**, **21b** and **5a** showed a strong inhibitory effect (15–25 mm) against *Escherichia coli,* as an example of Gram-negative bacteria. While the others showed a weak to a moderate inhibition effect against *Pseudomonas aeruginosa*, *Proteus* sp. The antifungal activities are presented in Table 1. In case of unicellular fungi most of the compounds showed a strong antifungal effect against *Candida albicans*. These compounds are **22a**, **25b**, **21a**, **9c** and **19b** while the other compounds were characterized by moderate antifungal effect. In the case of filamentous fungi, compounds **22a**, **25b**, **4b**, **5a**, **5b**, **9c**, **19d**, **22b** and **24a** have a higher antifungal effect against *Aspergillus niger* and a moderate activity against *A. flavus* in comparison with the antifungal standard antibiotic (Neomycin) used in this study. Finally, the demonstration of the activity of compound **25b** against gram-positive bacteria, gram-negative bacteria, and fungi is an indication that this compound can be used in the treatment of the tested pathogens due to its broad spectrum effect.

The structure-activity relationships (SAR) of these synthesized compounds (Tetracyclic derivatives) may be due to their ability for inhibiting the cell growth by inhibiting the protein synthesis [17,18].

The minimum inhibitory concentration (MIC) of the synthesized compounds is presented in the Table 2. The MIC varied from 50 to 500 µg/mL based on the tested compounds. The MIC of the compound **25b** was 50, 50, 75 and 100 µg/mL against tested pathogens *E. coli*, *C. albicans*, *B. subtilis* and *A. niger*. On the other hand, the MIC of the compound **22a** was 100 µg/mL against *Bacillius sutbtili*s, while the MIC of the compound **9c** was 200 µg/mL against *Bacillius sutbtilis*. Finally, we can interpret that some of samples have strongest antifungal and antibacterial activities. The demonstration of the activity against gram-positive bacteria, gram-negative bacteria, and fungi is an indication that the compounds have a broad spectrum effect. A few of the test compounds possessed a broad spectrum of activity having MIC values ranging from 50 to 200 µg/mL.

**Table 1:** Antimicrobial activity of compounds at 10 mg/mL.

Comp. No.	Bacteria						Fungi		
	G + ve			G − ve			unicellular	filamentous	
	*B. subtilits*	*S. aureus*	*Ent. faecalis*	*E. coli*	*P. aeruginosa*	*Proteus* sp.	*C. albicans*	A. niger	*A. flavus*
**4a**	17	17	14	17	14	15	15	12	11
**4b**	15	15	12	15	12	13	00	20	18
**5a**	00	18	15	18	15	14	15	20	19
**5b**	00	12	11	12	11	12	15	20	15
**9a**	12	00	00	00	00	00	00	00	00
**9b**	12	00	00	00	00	00	00	00	00
**9c**	22	20	17	20	18	17	17	20	16
**16a**	20	16	14	16	14	15	00	00	00
**16b**	12	00	00	00	00	00	00	00	00
**16c**	15	15	13	15	12	13	00	00	00
**16d**	14	13	12	13	12	11	00	00	00
**19a**	12	00	00	00	00	00	00	00	00
**19b**	22	20	17	20	17	18	17	00	00
**19c**	12	00	00	00	00	00	00	00	00
**19d**	20	17	14	17	14	15	00	20	17
**21a**	17	15	13	15	12	13	20	00	00
**21b**	14	20	18	20	15	17	00	00	00
**22a**	25	00	00	00	00	00	30	40	20
**22b**	15	00	00	00	00	00	15	18	16
**24a**	15	00	00	00	00	00	15	18	15
**24b**	12	00	00	00	00	00	15	00	00
**25a**	17	00	00	00	00	00	00	00	00
**25b**	27	25	19	25	19	20	25	29	20
S * =10 µg	14	00	00	00	00	00	12	00	00
TE * =30 µg	10	00	00	00	00	00	23	00	00
N * =30 µg	00	00	00	00	00	00	00	16	14
T * =30 µg	30	00	00	00	00	00	00	00	00

* S = Streptomycin; TE = Tetracycline; N = Neomycin and T = Oxytetracyciline.

**Table 2:** Minimum inhibitory concentration (MIC) of different compounds against of tested pathogens.

Comp. No.	Inhibition Zone Diameters (mm)				MIC (µg/mL)			
	*B. sutbtilis*	*E. coli*	*C. albicans*	*A. niger*	*B. sutbtilis*	*E. coli*	*C. albicans*	*A. niger*
**25b**	11	11	11	11	75	50	50	100
**22a**	11	-	-	-	100	-	-	-
**9c**	11	-	-	-	200	-	-	-

## 3. Experimental Section

### 3.1. Chemistry

All melting points were measured on a Gallenkamp melting point apparatus (Weiss Gallenkamp, London, UK). The infrared spectra were recorded in potassium bromide disks on a PyeUnicam SP 3300 and Shimadzu FT IR 8101 PC infrared spectrophotometers (PyeUnicam Ltd. Cambridge, UK and Shimadzu, Tokyo, Japan, respectively). The NMR spectra were recorded on a Varian Mercury VX-300 NMR spectrometer (Varian, Palo Alto, CA, USA). ^1^H spectra were run at 300 MHz and ^13^C spectra were run at 75.46 MHz in deuterated chloroform (CDCl_3_) or dimethyl sulfoxide (DMSO-*d*_6_). (Sigma-Aldrich, St. Louis, MO, USA). Chemical shifts are given in parts per million and were related to that of the solvent. Mass spectra were recorded on a Shimadzu GCMS-QP 1000 EX mass spectrometer (Shimadzu, Tokyo, Japan) at 70 eV. Elemental analyses were carried out at the Micro-analytical Centre of Cairo University, Giza, Egypt and recorded on Elementar-Vario EL (ELTRA GmbH, Haan, Germany) automatic analyzer. Compounds **1b**, **14a**–**c** and **18a**–**c** were prepared by following the reported procedures in the literature [19,20,21,22]. The *in vitro* antimicrobial screening was performed by Chemistry of Natural and Microbial Products Dept., National Research Centre, Cairo-12622, Cairo, Egypt.

#### 3.1.1. Preparation of the Thioamide Derivatives **4a**,**b**

A solution of finely ground KOH (0.12 g, 2 mmol) and benzosuberone derivatives **1a**,**b** (2 mmol) in DMF (10 mL), was stirred for 2 h. Phenyl isothiocyanate (0.27 g, 10.0 mmol) was then added drop-wise and the mixture was stirred for 10–12 h. The mixture was poured onto cold water acidified with 1*N* HCl. the solid product obtained was filtered off, washed with water, dried, and finally recrystallized with the prober solvent to afford the thioamidederivatives **4a**,**b**.

*6,7,8,9-Tetrahydro-6-(mercapto(phenylamino)methylene)benzo[7]annulen-5-one* **4a**. Yield: (0.24 g, 81%); mp: 185–187 °C; as a pale yellow crystals (MeOH). IR (KBr, cm^−1^) *v* 3430 (NH), 1637 (C=O). ^1^H-NMR (DMSO-*d*_6_): δ 1.90 (m, 2H, CH_2_), 2.41–3.20 (m, 4H, 2CH_2_), 6.99–7.90 (m, 9H, Ar-H), 8.50 (br s, 1H, NH, D_2_O exchangeable). MS *m/z* (%): 296 [M + 1]^+^ (5), 295 [M]^+^ (25), 219 (10), 205 (40), 160 (30), 92 (100), 77 (50). Anal. Calcd. for C_18_H_17_NOS (295.40): C, 73.19; H, 5.80; N, 4.74; S, 10.85. Found: C, 73.44; H, 5.89; N, 4.62; S, 10.79.

*6,7,8,9-Tetrahydro-6-(mercapto(phenylamino)methylene)-3-nitrobenzo[7]annulen-5-one* **4b**. Yield: (0.27 g, 79%); mp: 215–217 °C; yellow crystals (EtOH). IR (KBr, cm^−1^): *v* 3433 (NH), 1663 (C=O). ^1^H-NMR (DMSO-*d*_6_): δ 1.94–3.30 (m, 6H, 3CH_2_), 7.01–8.27 (m, 8H, Ar-H), 9.20 (br s, 1H, NH, D_2_O exchangeable). ^13^H-NMR (DMSO-*d*_6_): δ 25.41, 25.60, 39.90, 115.68, 119.98, 120.39, 124.39, 129.51, 130.88, 138.88, 139.05, 142.92, 147.15, 155.41, 189.93. MS *m/z* (%): 341 [M + 1]^+^ (3), 340[M]^+^ (15), 338 (100), 262 (35), 205 (60). Anal. Calcd. for C_18_H_16_N_2_O_3_S (340.40): C, 63.51; H, 4.74; N, 8.23; S, 9.42. Found: C, 63.44; H, 4.69; N, 7.97; S, 9.48.

#### 3.1.2. General Procedure for Preparation of the S-Methylated Thioamide Derivatives **5a**,**b**

To a stirred solution of the thioamide derivatives **4a**,**b** (1 mmol) and potassium carbonate (0.14 g, 1 mmol) in DMF (10 mL), iodomethane (0.28 g, 2 mmol) was added and stirring was continued for another 12 h. The mixture was poured onto crushed ice and the solid product obtained was filtered off, washed with water, dried, and finally recrystallized from the prober solvent to afford colorless crystals of compounds **5a**,**b**.

*6,7,8,9-Tetrahydro-6-(methylthio(phenylamino)methylene)benzo[7]annulen-5-one* **5a**. Yield: (0.26 g, 84%); mp: 195–197 °C; buff powder (MeOH/dioxan). IR (KBr, cm^−1^): *v* 3400(NH), 1674 (C=O). ^1^H-NMR (DMSO-*d*_6_): δ 1.77–2.51 (m, 6H, 3CH_2_), 3.08 (s, 3H, CH_3_), 6.86–7.79 (m, 9H, Ar-H), 8.31 (br s, 1H, NH, D_2_O exchangeable). MS *m/z* (%): 310 [M + 1]^+^ (8), 309 [M]^+^ (45), 261 (30), 217 (20), 115 (70), 91 (50), 77 (100). Anal. Calcd. for C_19_H_19_NOS (309.43): C, 73.75; H, 6.19; N, 4.53; S, 10.36. Found: C, 73.52; H, 6.10; N, 4.62; S, 10.44.

*6,7,8,9-Tetrahydro-6-(methylthio(phenylamino)methylene)-3-nitrobenzo[7]annulen-5-one* **5b**. Yield: (0.31 g, 84%); mp: 225–227 °C; pale yellow crystals (EtOH/DMF). IR (KBr, cm^−1^): *v* 3430 (NH), 1664 (C=O). ^1^H-NMR (DMSO-*d*_6_): δ 1.77 (m, 2H, CH_2_), 2.30–2.51 (m, 4H, 2CH_2_), 3.30 (s, 3H, CH_3_), 7.18–8.57 (m, 8H, Ar-H), 9.01 (br s, 1H, NH, D_2_O exchangeable). MS *m/z* (%): 355 [M + 1]^+^ (5), 354 [M]^+^ (25), 205 (35), 159 (40), 93 (100), 77(70). Anal. Calcd. for C_19_H_18_N_2_O_3_S (354.42): C, 64.39; H, 5.12; N, 7.90; S, 9.05. Found: C, 64.26; H, 5.03; N, 7.78; S, 9.14.

#### 3.1.3. Reaction of Thioamide Derivatives **4** with α-Halo Carbonyl Compounds: General Procedure for the Preparation of **9a**–**c**

*Method A*: To a solution of the thioamide derivatives **4a** and **4b** (1 mmol) and 1 mmol of chloroacetone **6** or phenacyl chloride **7** in EtOH (10 mL), 0.2 mL of triethylamine were added. The reaction mixture was refluxed for 10–15 h and then allowed to cool. The solid product obtained was filtered off, washed with EtOH, dried, and finally recrystallized from the prober solvent to afford the corresponding thiophenes **9a**–**c**, respectively.

By the same method, **4a** (1 mmol) was reacted with 3-chloroacetyl acetone (1 mmol) to afford **9a**, which is identical in all respects (mp, TLC and spectra) in comparison with an authentic sample that obtained from the reaction of **4a** and chloroacetone. 

*Method B*: To a mixture of the thioamide derivatives **4a**,**b** (1 mmol) and chloroacetone **6** or phenacyl chloride **7** (1 mmol) in DMF (5 mL), 0.19 g potassium carbonate was added. The reaction mixture was stirred at ambient temperature for 10 h, and then poured onto ice cold water acidified with 1*N* HCl. The solid product obtained was filtered off, washed with water, dried and finally recrystallized from the prober solvent to afford products identical in all respect with compounds **9a**–**d**.

*(2-Acetyl-5-phenylimimno)thiophen[c,f]benzo[7]anulene* **9a**. Yield: (0.29 g, 87%); mp: 187–190 °C; buff powder (MeOH). IR (KBr, cm^−1^): *v* 3430 (NH), 1691(C=O). ^1^H-NMR (DMSO-*d*_6_): δ 1.72 (m, 2H, CH_2_), 2.15–2.64 (m, 4H, 2CH_2_), 2.80 (s, 3H, CH_3_), 6.97–7.59 (m, 9H, Ar-H), 8.60 (s, 1H, NH, D_2_O exchangeable). MS *m/z* (%): 334 [M + 1]^+^ (7), 333 [M]^+^ (35), 308 (50), 293 (15), 194 (20), 118 (100), 92 (15), 77 (40). Anal. Calcd. for C_21_H_19_NOS (333.45): C, 75.64; H, 5.74; N, 4.20; S, 9.62. Found: C, 75.32; H, 5.61; N, 4.05; S, 9.74.

*(2-Acetyl-5-phenylimino)thiophen[c,f]-3-nitrobenzo[7]anulene* **9b**. Yield: (0.30 g, 80%); mp: 200–202 °C; yellow crystals (EtOH). IR (KBr, cm^−1^): *v* 3428 (NH), 1654 (C=O). ^1^H-NMR (DMSO-*d*_6_): δ 1.72 (m, 2H, CH_2_), 2.35–2.591 (m, 4H, 2CH_2_), 3.30 (s, 3H, CH_3_), 6.68–8.72 (m, 8H, Ar-H), 9.53 (s, 1H, NH, D_2_O exchangeable). ^13^H-NMR (DMSO-*d*_6_): δ 21.35, 28.23, 34.12, 39.45, 116.30, 119.35, 122.21, 123.10, 125.50, 129.60, 135.90, 136.10, 138.50, 141.33, 143.31, 145.21, 150.13, 187.21. MS *m/z* (%): 379 [M + 1]^+^ (4), 378 [M]^+^ (25), 343 (10), 258 (40), 212 (100), 200 (35), 142 (70), 91 (65), 77 (60). Anal. Calcd. for C_21_H_18_N_2_O_3_S (378.44): C, 66.65; H, 4.79; N, 7.40; S, 8.47. Found: C, 66.51; H, 4.73; N, 7.47; S, 8.55.

*(2-Benzoyl-5-phenylimino)thiophen[c,f]benzo[7]anulen* (**9c**). Yield: (0.31 g, 81%); mp: 195–198 °C; yellow powder (EtOH). IR (KBr, cm^−1^): *v* 3425 (NH), 1681 (C=O). ^1^H-NMR (DMSO-*d*_6_): δ 1.82–2.84 (m, 6H, 3CH_2_), 6.97–7.59 (m, 14H, Ar-H), 8.60 (s, 1H, NH, D_2_O exchangeable). MS *m/z* (%): 396 [M + 1]^+^ (10), 395 [M]^+^ (45), 303 (25), 293 (15), 199 (30), 115 (100), 92 (77), 77 (40). Anal. Calcd. for C_26_H_21_NOS (395.52): C, 78.95; H, 5.35; N, 3.54; S, 8.11. Found: C, 78.84; H, 5.28; N, 3.62; S, 8.15.

#### 3.1.4. Reactions of Thioamide Derivatives **4a**,**b** with *C*-Acetyl-*N*-arylhydrazonoyl Chlorides **14a**,**b** and *C*-Ethoxycarbonyl-*N*-arylhydrazonoyl chlorides **18a**,**b**

The reactions of the thioamide derivatives **4a** and **4b** with hydrazonoyl chlorides **14a**,**b** and/or **18a**,**b**, were carried out as described above for the synthesis of thiophene derivatives (method A), to afford the corresponding 1,3,4-thiadiazol derivatives **16a**–**d** and **19a**–**d**, respectively.

*6-(5-Acetyl-3-p-tolyl-1,3,4-thiadiazol-2(3H)-ylidene)-6,7,8,9-tetrahydrobenzo[7]-annulen-5-one* **16a**. Yield: (0.3 g, 79%); mp: 225–227 °C; yellow powder (MeOH/dioxan). IR (KBr, cm^−1^): *v* 1668, 1653 (C=O). ^1^H-NMR (DMSO-*d*_6_): δ 1.57 (m, 2H, CH_2_), 2.40–2.46 (m, 4H, 2CH_2_), 3.10 (s, 3H, CH_3_), 3.43 (s, 3H, CH_3_), 7.10–7.50 (m, 8H, Ar-H). MS *m/z* (%): 377 [M + 1]^+^ (9), 376 [M]^+^ (75), 187 (45), 160 (20), 91 (80), 86 (100). Anal. Calcd. for C_22_H_20_N_2_O_2_S (376.47): C, 70.19; H, 5.35; N, 7.44; S, 8.52 . Found: C, 69.98; H, 5.19; N, 7.66; S, 8.47.

*6-(5-Acetyl-3-p-tolyl-1,3,4-thiadiazol-2(3H)-ylidene)-6,7,8,9-tetrahydro-3-nitrobenzo[7]-annulen-5-*one **16b**. Yield: (0.38 g, 90%); mp: 260–262 °C; brown crystals (EtOH/dioxan). IR (KBr, cm^−1^): *v* 1690, 1645 (C=O). ^1^H-NMR (DMSO-*d*_6_): δ 1.70 (m, 2H, CH_2_), 2.40–2.46 (m, 4H, 2CH_2_), 3.10 (s, 3H, CH_3_), 3.43 (s, 3H, CH_3_), 7.30–7.90 (m, 7H, Ar-H). MS *m/z* (%): 423 [M + 2]^+^ (11), 421 [M]^+^ (55), 287 (15), 203 (30), 159 (20), 91 (35). Anal. Calcd. For C_22_H_19_N_3_O_4_S (421.47): C, 62.69; H, 4.54; N, 9.97; S, 7.61. Found: C, 62.35; H, 4.39; N, 10.14; S, 7.41.

*6-(5-Acetyl-3-(4-fluorophenyl)-1,3,4-thiadiazol-2(3H)-ylidene)-6,7,8,9-tetrahydrobenzo[7]-annulen-5-one* **16c**. Yield: (0.32 g, 85%); mp: 240–242 °C; gray powder (dioxan). IR (KBr, cm^−1^): *v* 1671, 1653 (C=O). ^1^H-NMR (DMSO-*d*_6_): δ 1.53 (m, 2H, CH_2_), 2.38–2.46 (m, 4H, 2CH_2_), 3.43 (s, 3H, CH_3_), 6.76–7.40 (m, 8H, Ar-H). ^13^H-NMR (DMSO-*d*_6_): δ 23.13, 23.80, 25.41, 38.90, 116.44, 117.60, 119.90, 126.23, 128.75, 130.67, 134.28, 136.13, 140.35, 145.24, 151.20, 154.31, 187.23, 195.25.MS *m/z* (%): 381 [M + 1]^+^ (9), 380 [M]^+^ (45), 187 (25), 160 (27), 91 (85), 86 (100). Anal. Calcd. For C_21_H_17_N_2_O_2_S (380.44): C, 66.30; H, 4.50; N, 7.36; S, 8.43. Found: C, 66.11; H, 4.37; N, 7.55; S, 8.64.

*6-(5-Acetyl-3-(4-fluorophenyl)-1,3,4-thiadiazol-2(3H)-ylidene)-6,7,8,9-tetrahydro-3-nitrobenzo-[7]annulen-5-one* **16d**. Yield: (0.35 g, 87%); mp: 275–277 °C; green crystals (EtOH/DMF). IR (KBr, cm^−1^): *v* 1680, 1655 (C=O). ^1^H-NMR (DMSO-*d*_6_): δ 1.57 (m, 2H, CH_2_), 2.03–2.56 (m, 4H, 2CH_2_), 3.43 (s, 3H, CH_3_), 6.76–8.40 (m, 7H, Ar-H). MS *m/z* (%): 425 [M]^+^ (30) 245 (45), 171 (20), 95 (100), 77 (60). Anal. calcd. for C_21_H_16_N_3_O_4_S (425.43): C, 59.29; H, 3.79; N, 9.88; S, 7.54. Found: C, 59.09; H, 3.67; N, 10.17; S, 7.73.

*Ethyl 4,5-Dihydro-5-(5,6-dihydro-9-oxo-5H-benzo[7]annulen-8(7H)-ylidene)-4-phenyl-1,3,4-thiadiazole-2-carboxylate* **19a**. Yield: (0.31 g, 79%); mp: 218–220 °C; brown crystals (EtOH/DMF). IR (KBr, cm^−1^): *v* 1718, 1675 (C=O). ^1^H-NMR (DMSO-*d*_6_): δ 1.71 (m, 2H, CH_2_), 2.36–2.80 (m, 6H, 3CH_2_), 4.2 (q, *J* = 7.1 Hz, 2H, CH_3_), 6.76–7.20 (m, 9H, Ar-H), ^13^H-NMR (DMSO-*d*_6_): δ 13.80, 21.35, 23.85, 35.55, 60.25, 116.30, 118.82, 126.62, 128.70, 129.60, 130.60, 133.00, 134.72, 140.40, 141.80, 154.50, 161.12, 178.35. MS *m/z* (%): 393 [M + 1]^+^ (6), 392 [M]^+^ (25), 346 (20), 194 (15), 242 (45), 150 (30), 92 (100), 77 (80), 65 (60). Anal. Calcd. for C_22_H_20_N_2_O_3_S (392.47): C, 67.33; H, 5.14; N, 7.14; S, 8.17. Found: C, 67.12; H, 4.98; N, 7.35; S, 8.34.

*Ethyl 4,5-Dihydro-5-(6,7-dihydro-2-nitro-9-oxo-5H-benzo[7]annulen-8(9H)-ylidene)-4-phenyl-1,3,4-thiadiazole-2-carboxylate* **19b**. Yield: (0.33 g, 75%); mp: 227–229 °C; buff crystals (EtOH/dioxan). IR (KBr, cm^−1^): *v* 1722, 1638 (C=O). ^1^H-NMR (DMSO-*d*_6_): δ 1.50 (t, *J* = 7.0 Hz, 3H, CH_3_), 1.71–2.91 (m, 6H, 3CH_2_), 4.16 (q, *J* = 7.1 Hz, 2H, CH_2_), 6.83–7.49 (m, 8H, Ar-H), ^13^H-NMR (DMSO-*d*_6_): δ 21.11, 23.15, 40.33, 46.39, 64.90, 125.44, 127.60, 127.90, 128.23, 128.75, 133.67, 136.28, 139.13, 141.35, 196.24. MS *m/z* (%): 438 [M + 1] (6), 437 [M]^+^ (30), 392 (20), 364 (15), 288 (35), 203 (50), 125 (100), 77 (60). Anal. Calcd. for C_22_H_19_N_3_O_5_S (437.47): C, 60.40; H, 4.38; N, 9.61; S, 7.33. Found: C, 59.88; H, 4.26; N, 9.85; S, 7.51.

*Ethyl 4,5-Dihydro-5-(5,6-dihydro-9-oxo-5H-benzo[7]annulen-8(7H)-ylidene)-4-p-tolyl-1,3,4-thiadiazole-2-carboxylate* **19c**. Yield: (0.29 g, 71%); mp: 192–195 °C; gray crystals (EtOH/DMF). IR (KBr, cm^−1^): *v* 1715, 1661 (C=O). ^1^H-NMR (DMSO-*d*_6_): δ 1.30 (t, *J* = 6.9 Hz, 3H, CH_3_), 2.23 (s, 3H, CH_3_), 2.46–2.92 (m, 4H, 2CH_2_), 3.53 (q, *J* = 7.1 Hz, 2H, CH_2_), 4.27 (m, 2H, CH_2_), 6.76–7.50 (m, 8H, Ar-H). MS *m/z* (%): 407 [M + 1]^+^ (4), 406 [M]^+^ (15), 333 (25), 315 (100), 242 (45), 158 (30), 91 (50). Anal. calcd. for C_23_H_22_N_2_O_3_S (406.50): C, 67.96; H, 5.46; N, 6.89; S, 7.89. Found: C, 67.52; H, 5.28; N, 7.06; S, 8.13.

*Ethyl 4,5-Dihydro-5-(6,7-dihydro-3-nitro-9-oxo-5H-benzo[7]annulen-8(9H)-ylidene)-4-p-tolyl-1,3,4-thiadiazole-2-carboxylate* **19d**. Yield: (0.37 g, 82%); mp: 235–2375 °C; pale yellow crystals (EtOH/DMF). IR (KBr, cm^−1^): *v* 1735, 1670 (C=O). ^1^H-NMR (DMSO-*d*_6_): δ 1.35 (t, *J* = 6.9 Hz,3H,CH_3_), 1.91–2.61 (m, 6H, 3CH_2_), 3.28 (s, 3H, CH_3_), 4.37 (q, *J* = 7.1 Hz, 2H, CH_2_), 7.22–8.28 (m, 7H, Ar-H), ^13^H-NMR (DMSO-*d*_6_): δ 13.35, 23.55, 25.90, 26.11, 38.23, 61.25, 116.32, 121.23, 125.75, 126.61, 127.67, 129.22, 135.13, 141.35, 143.43, 148.20, 148.60, 155.62, 162.20, 185.32. MS *m/z* (%): 452 [M + 1]^+^ (12), 451 [M]^+^ (50), 360 (15), 234 (61), 199 (36), 125 (29), 77 (100). Anal. calcd. for C_23_H_21_N_3_O_5_S (451.49): C, 61.01; H, 4.43; N, 9.54; S, 7.10. Found: C, 61.18; H, 4.69; N, 9.31; S, 7.31.

#### 3.1.5. General Procedure for the Preparation of Compounds **21a**,**b**

A mixture of the **1a**,**b** (5 mmol), mercaptoacetic acid (5 mmol) and *p*-tolunesulfonic acid (PTSA) in dry benzene (50 mL) was refluxed for 72 h and allowed to cool to room temperature then diluted with water (30 mL). The mixture was washed with saturated NaHCO_3_ followed by 0.1*N* HCl solution. The organic layer was separated and dried over anhydrous sodium sulfate, then evaporated under reduced pressure. The solid products were collected by filtration, washed with EtOH, dried and recrystallized from the prober solvent to afford compounds **21a**,**b**.

*3-((E)-6,7-Dihydro-5H-benzo[7]annulen-9-ylthio)propanoic acid* **21a**. Yield: (0.21 g, 84%); mp: 193–195 °C; White crystals (MeOH). IR (KBr, cm^−1^): *v* 1722 (C=O). ^1^H-NMR (DMSO-*d*_6_): δ 1.65 (m, 2H, CH_2_), 1.96 (t, *J* = 6.7 Hz, 2H, CH_2_), 2.55–3.14 (m, 6H, 3CH_2_), 7.10–7.50 (m, 5H, Ar-H + CH=C), 11.5 (s, 1H, OH). MS *m/z* (%): 249 [M + 1]^+^ (25), 248 [M]^+^ (100), 175 (36), 143 (75). Anal. Calcd. for C_14_H_16_ O_2_S (248.34): C, 67.71; H, 6.49; S, 12.91. Found: C, 67.55; H, 6.33; S, 13.17.

*3-((E)-6,7-Dihydro-3-nitro-5H-benzo[7]annulen-9-ylthio)propanoic acid* **21b**. Yield: (0.35 g, 86%); mp: 208–210 °C; pale yellow crystals (EtOH). IR (KBr, cm^−1^): *v* 1735 (C=O). ^1^H-NMR (DMSO-*d*_6_): δ 1.65 (m, 2H, CH_2_), 1.69 (t, *J* = 6.9 Hz, 2H, CH_2_), 2.55–2.90 (m, 4H, 2CH_2_), 3.14 (t, *J* = 7.0 Hz, 2H, CH_2_), 7.33–8.50 (m, 4H, Ar-H + CH=C), 12.3 (s, 1H, OH). MS *m/z* (%): 294[M + 1]^+^ (25), 293[M]^+^ (100), 220 (70), 188 (40), 143 (36). Anal. Calcd. for C_14_H_15_NO_4_S (293.34): C, 57.32; H, 5.15; N, 477; S, 10.93. Found: C, 57.44; H, 5.04; N, 4.87; S, 11.16.

#### 3.1.6. General Procedure for the Preparation of Compounds **22a**,**b**

To a solution of the appropriate mercaptopropanoic acid derivatives **21a**,**b** in benzene (thiophene free) (100 mL), phosphorus pentoxide (1 mmol) was added. The resulting mixture was refluxed for 72 h and allowed to cool to room temperature then diluted with water (30 mL), and washed with a saturated NaHCO_3_ solution, then we separated the water and HCl was added. The solid products that formed were collected by filtration, washed with water, dried, and recrystallized from the ethanol to afford the newly tricyclic compounds **22a**,**b**.

*2,3-Dihydrothiopyran-4-one[b,f]-benzo[7]-9,10,11-trihydroanulene* **22a**. Yield: (0.20 g, 86%); mp: 210–212 °C; buff powder (EtOH). IR (KBr, cm^−1^): *v* 1695 (C=O). ^1^H-NMR (DMSO-*d_6_*): δ 2.05 (m, 2H, CH_2_), 2.26–2.50 (m, 4H, 2CH_2_), 2.80 (m, 2H, CH_2_), 3.41 (m, 2H, CH_2_), 7.01–7.50 (m, 4H, Ar-H).MS *m/z* (%): 231 [M + 1]^+^ (25), 230 [M]^+^ (100), 214 (36), 142 (75). Anal. Calcd. for C_14_H_14_OS (230.33): C, 73.01; H, 6.13; S, 13.92. Found: C, 72.79; H, 5.95; S, 14.26.

*2,3-Dihydrothiopyran-4-one[b,f]-3-nitrobenzo[7]9,10,11-trihydroanulene* **22b**. Yield: (0.23 g, 83%); mp: 227–230 °C; gray crystals (Dioxane). IR (KBr, cm^−1^): *v* 1683 (C=O). ^1^H-NMR (DMSO-*d*_6_): δ 2.15–2.70 (m, 6H, 3CH_2_), 3.25 (m, 2H, CH_2_), 4.07 (t, *J* = 7.0 Hz, 2H, CH_2_), 7.71–8.47 (m, 3H, Ar-H).). ^13^H-NMR (DMSO-*d*_6_): δ 23.50, 25.9, 28.80, 39.20, 38.50, 120.40, 122.50, 123.99, 130.60, 135.55, 145.01, 148.60, 151.7, 198.90. MS *m/z* (%): 276 [M + 1]^+^ (23), 275 [M]^+^ (100), 219 (70), 186 (40), 115 (36), 63 (29). Anal. calcd. for C_14_H_13_NO_3_S (275.32): C, 61.07; H, 4.76; N, 5.09; S, 11.65. Found: C, 60.93; H, 4.63; N, 5.21; S, 11.89.

#### 3.1.7. General Procedure for the Preparation of Compounds **24a**,**b**

A mixture of the compounds **22a** and/or **22b** (2 mmol), mercaptoacetic acid (2 mmol) and PTSA (2 mmol) in dry benzene (100 mL) was refluxed for 24 h and allowed to cool to room temperature. Phosphorus pentoxide (1 mmol) was then added and the resulting mixture was refluxed for additional 5 h and allowed to cool. The mixture was then diluted with water (30 mL), washed by saturated NaHCO_3_ and 0.1*N* HCl. The organic layer was separated and dried over anhydrous sodium sulfate, then evaporated under reduced pressure. The solid products that formed were collected by filtration, washed with water, dried, and recrystallized from the prober solvent to afford compounds **24a**,**b**.

*2,3-Dihydrothiopyrano[3,2-b]thiopyran-4(8H)-one[d,f]benzo[7]anulen* **24a**. Yield: (0.23 g, 86%); mp: 280–282 °C; white crystal (EtOH/Dioxan). IR (KBr, cm^−1^): *v* 1692 (C=O). ^1^H-NMR (DMSO-*d*_6_): δ 2.25–2.50 (m, 6H, 3CH_2_), 3.25–3.38 (m, 4H, 2CH_2_), 3.73 (s, 2H, CH_2_), 7.30–7.57 (m, 4H, Ar-H). ^13^H-NMR (DMSO-*d*_6_): δ 24.1, 24.9, 35.50, 38.40, 38.90, 126.00, 126.7, 128.01, 128.7, 132.0, 135.17, 138.99, 196.03.MS *m/z* (%): 301 [M + 1]^+^ (23), 300 [M]^+^ (100), 211 (50), 142 (35). Anal. calcd. for C_17_H_16_OS_2_ (300.44): C, 67.96; H, 5.37; S, 21.35. Found: C, 67.84; H, 5.26; S, 21.66.

*2,3-Dihydrothiopyrano[3,2-b]thiopyran-4(8H)-one[d,f]-3-nitrobenzo[7]anulene* **24b**. Yield: (0.31 g, 90%); mp: >300 °C; brown crystals (EtOH/DMF). IR (KBr, cm^−1^): *v* 1698 (C=O). ^1^H-NMR (DMSO-*d*_6_): δ 2.30–2.78 (m, 6H, 3CH_2_), 3.27 (t, *J* = 6.4 Hz, 2H, CH_2_), 3.33 (t, *J* = 6.8 Hz, 2H, CH_2_), 3.89 (s, 2H, CH_2_), 7.64–8.37 (m, 3H, Ar-H). MS *m/z* (%): 346 [M + 1]^+^ (19), 345 [M]^+^ (100), 299 (15), 257 (40), 209 (20), 60 (25). Anal. Calcd. for C_17_H_15_NO_3_S_2_ (345.44): C, 59.11; H, 4.38; N, 4.05; S, 18.56. Found: C, 58.97; H, 4.26; N, 4.15; S, 18.86.

#### 3.1.8. General Procedure for the Preparation of Compounds **25a**,**b**

A mixture of the compound **24a** and/or **24b** (1 mmol) and hydrogen peroxide (2 mmol) was refluxed for 24 h and allowed to cool to room temperature then diluted with water (30 mL). The solid products that formed were collected by filtration, washed with water, dried and recrystallized from the ethanol to afford compounds **25a**,**b**.

*2,3-Dihydro-dioxothiopyrano[3,2-b]dioxothiopyran-4(8H)-one[d,f]benzo[7]anulen* **25a**. Yield (0.23 g, 65%); mp: > 300 °C; gray crystals (Dioxan). IR (KBr, cm^−1^): *v* 1689 (C=O). ^1^H-NMR (DMSO-*d*_6_): δ 1.76 (m, 2H, CH_2_), 2.15–2.30 (m, 4H, 2CH_2_), 2.63 (s, 2H, CH_2_), 3.27–3.52 (m, 4H, 2CH_2_), 7.01–7.47 (m, 4H, Ar-H). MS *m/z* (%): 366 [M + 2]^+^ (3), 364 [M]^+^ (25), 246 (30), 142 (70), 115 (100). Anal. Calcd. for C_17_H_16_O_5_S_2_ (364.44): C, 56.03; H, 4.43; S, 17.60. Found: C, 55.76; H, 4.28; S, 18.04.

*2,3-Dihydro-dioxothiopyrano[3,2-b]dioxothiopyran-4(8H)-one[d,f]-3-nitrobenzo[7]anulene* **25b**. Yield (0.30 g, 75%); mp: > 300 °C; buff crystals (Dioxan). IR (KBr, cm^−1^): *v* 1695 (C=O). ^1^H-NMR (DMSO-*d*_6_): δ 1.90 (m, 2H, CH_2_), 2.15–2.30 (m, 4H, 2CH_2_), 2.53 (s, 2H, CH_2_), 3.27–4.09 (m, 4H, 2CH_2_), 7.71–8.47 (m, 3H, Ar-H). MS *m/z* (%): 410 [M + 1]^+^ (10), 409 [M]^+^ (100), 289 (15), 187 (40). Anal. Calcd. for C_17_H_15_NO_7_S_2_ (409.43): C, 49.87; H, 3.69; N, 3.42; S, 15.66. Found: C, 49.63; H, 3.57; N, 3.52; S, 15.89.

### 3.2. Biological Evaluation

#### 3.2.1. Antimicrobial Activity

The ability to inhibit the growth of Gram-positive and Gram-negative bacteria, yeasts and filamentous fungi was observed using an overlay method [17].

##### Sample Preparation

All samples were dissolved in dimethyl sulfoxide (RFCL Limited, New Delhi, India) DMSO at 10 mg/mL concentration as shown in the Table 1 in comparison with different standard antibiotics. Antibiotic discs of Streptomycin (S) (10 μg), Oxytetracycline (T) (30 μg) and Tetracyciline (TE) (30 μg) were used as positive controls for bacteria. Neomycin (N) (30 μg), was used for fungi. The experiment was performed in triplicate.

##### Strains and Media Used

The common pathogenic and food spoilage microorganisms were selected for their relevance in bakery products and other food: the gram-positive bacteria; *Bacillus subtilits* NRRL-B-4219, *Staphylococcus aureus* ATCC 6538, *Enterococcus faecalis* ATCC 19433 and the gram negative bacteria; *Escherichia coli* ATCC 25922, *Pseudomonas aeruginosa* ATCC 9027, *Proteus* sp., yeasts such as *Candida albicans* ATCC 10231, fungi *Aspergillus niger* NRRL 2766 (equivalent to ATCC16888), and *Aspergillus flavus* ATCC 16883.

The bacteria were slanted on nutrient agar (Merck, Darmstadt, Germany), Yeast was slanted and mentioned on Sabaroud’s agar medium (Lab M., Bury, Lancashire, UK) and the fungi was slanted and mentioned on the potato Dextrose Agar medium (Lab M Limited, Bury, Lancashire, UK). Mueller-Hinton agar (Lab M., Bury, Lancashire, UK) following the manufacturer’s instructions was used for the assay.

##### Bioassay

The antibacterial screening was essentially by the well diffusion agar method [23,24]. The organisms were streaked in radial patterns on the agar plates. Plates were incubated under aerobic conditions at 37 °C and 28 °C for 24 h and 48 h for bacteria and fungi, respectively. In order to obtain comparable results, all prepared solutions were treated under the same conditions under the same incubated plates. All tests were performed in triplicate. Plates were examined for evidence of antimicrobial activities, represented by a zone of inhibition of the microorganism's growth around the holes, and diameters of clear zones were expressed in millimeters [25].

#### 3.2.2. Determination of Minimum Inhibitory Concentration (MIC)

The *in vitro* minimum inhibitory concentration (MIC) of the synthesized compounds was determined by the agar well diffusion method. DMSO was used to prepare different concentrations ranging from 50 to 500 µg/mL by serial dilutions. The media were inoculated with 100 µL of each of the 10^6^ cfu/mL bacterial and fungal strains, and the assay was applied by an agar well diffusion method. Blank DMSO was used as negative control. The plates were incubated aerobically in an incubator at 37 °C for 24 h for bacterial strains and 25 °C for 48 h for fungal strains. The MIC was taken as the lowest concentration in the series dilution that prevented bacterial growth.

## 4. Conclusions

An efficient one step method for the synthesis of substituted thiophene derivatives **9** from thioamides **4** and **α-**haloketones has been performed. Reaction of **4** with *C*-acetyl-*N*-arylhydrazonoyl chlorides **14** and *C*-ethoxycarbonyl-*N*-arylhydrazonoyl chlorides **18** under basic conditions, afforded the corresponding 1,3,4-thiadiazoline derivatives **16** and **19**, respectively. Benzosuberones **1** underwent condensation followed by cyclization to obtain tricyclic thiopyran-4(5*H*)-one derivatives and tetracyclic ring systems **24** and **25**. The newly synthesized compounds showed to be active when tested as antimicrobial agents.
